# Dimensions of rivalry: China, the United States, and Europe

**DOI:** 10.1007/s42533-021-00065-z

**Published:** 2021-02-26

**Authors:** Volker Perthes

**Affiliations:** German Institute for International and Security Affairs (SWP), Berlin, Germany

**Keywords:** China, European Union, United States, Strategic autonomy, COVID-19, Multilateralism

## Abstract

The U.S.–China rivalry has become a guiding paradigm of international relation that can only be understood in its multidimensionality. Europe is not an equidistant observer of this conflict: European views of China have become much more critical, particularly with regard to human rights, China’s increasingly assertive regional posture and its perceived attempts to export its authoritarian political model. The COVID-19 pandemic has increased rather than mitigated mistrust. For Europe, the idea of “decoupling” from China is not an option. Rather Europe is developing its own instruments for a more coordinated China policy. It will likely continue to see China both as a competitor and as a multilateral partner and at the same time seek close coordination with the Biden administration on most international issues, including relations with China.

## Introduction^1^

The rivalry between the United States and China has become a guiding paradigm of international relations, shaping both strategic debates and real world political, military and economic dynamics. This does not mean that competition between Washington and Beijing—or even great power rivalries in general—determine all other international problems and conflicts. However, Sino-American competition is increasingly providing the framework through which actors throughout the world, not only the United States and China, view significant events and developments. The European Union (EU) and its member states have their own stakes in this rivalry. Similar to many other middle powers or regional organizations (ASEAN for example) with lesser economic and political weight, they refuse to submit to a bipolar logic that would force them to choose between an American and a Chinese economic or technological sphere. However, equidistance is not an option for the EU and its members. Instead, Europe is stepping up its efforts to define its own interests and priorities not only in geo-economic but also in geopolitical terms.

Since the end of the Cold War, nothing has had a more decisive impact on the structure of the international system than the rise of China and of Asia in general. Consequently, major powers have had to review and revise their strategies and approaches. The pivot to Asia by the Barack Obama administration (2008–2016) already reflected this changing landscape, aiming at repositioning the United States towards both the challenges and opportunities that shifts in economic, political and military power involved. While certainly not blind to power competition, the “pivot” was based on the assumption that a cooperative relationship with China was both desirable and possible.

Deeper strategic reviews and rethinking have taken place since about 2017. These changes coincided with the repositioning of U.S. foreign policies under the Donald Trump administration (2016–2021), but they were not solely caused by the idiosyncrasies of the new president. It was significant, though, that the U.S. government began to name China a “long-term strategic competitor” in its new National Security Strategy in 2017. Other countries also began to reconsider the framework through which they viewed China and relations with China. This included the European allies of the United States: in its London Declaration of December 2019, NATO spoke for the first time about challenges arising from China’s international weight and policies—but, notably, also about opportunities. The EU, also in 2019, tried to distinguish different aspects of its relationship with China, using four different terms to define the People’s Republic as a global cooperation partner, a negotiating partner, an economic competitor—and also as a “systemic rival” (European Commission 2019). At the same time, China’s political elite seemed—probably rightly so—increasingly convinced that the United States intended to contain the expansion of Chinese influence, or even China’s own development.

## A multidimensional conflict

Trade disputes have been in the limelight of the U.S.–China relationship, particularly during the Trump presidency. They are but one aspect of the rivalry, however, and by no means the most important one. Only if one understands the multidimensionality of the U.S.–Chinese conflict constellation will it be possible to find appropriate policy responses. Obviously, the global balance of power and the status of the two powers in the international system is one of the key issues (Yan 2019). President Donald Trump seemed to regard superiority, especially military dominance, as an end in itself and not primarily as a means to advance certain interests and values. President Xi Jinping is apparently driven by a vision of order “with Chinese characteristics” in which superiority is both a means and an end. But the competition between the rising power and the established superpower also has its own security, economic, technological and ideological dimensions. Acting personalities play a role too.

Overall, influence—on other states, regions, and societies—is at stake. From a Chinese perspective, America will never voluntarily grant China greater international influence. In the U.S., China is regarded as a revisionist power that is striving for global supremacy in the long term. More balanced positions exist in both countries but their influence on public discourse has been marginal, or marginalized.

At the same time, perceptions of the military threat posed by the other power are increasing in China and the U.S. A classic security dilemma is gradually developing: efforts by one state to strengthen its security reinforce the feeling of insecurity in the other. This is particularly true in the maritime domain. China is expanding its fleet to secure supply routes, expand influence, and prevent containment by American bases and allies. The U.S. in turn sees China’s growing naval capabilities as a threat to its own military bases and to its alliance system in the Indo-Pacific region.

Economic competition and conflicts over trade, economic, and financial policies form a real, distinct dimension of this rivalry. American criticism of unfair competition or breaches of rules by China is widely shared in Europe. Trade conflict is closely linked to global governance issues, which are of vital importance, especially from a European perspective. This applies, for example, to the future of binding, multilateral trade rules and international institutions. Importantly, and in contrast to the patterns of the past three to four decades, bilateral trade between the U.S. and China is no longer a stabilizer that balances out political conflicts. Rather, the rivalry between the two powers will continue to have a decisive impact on international politics—even if Washington and Beijing should one day conclude a comprehensive trade agreement. Even trade between Europe and China no longer smooths over political differences as it did in the past.

The technological dimension of the U.S.–China rivalry will also certainly survive any settlement of trade disputes. While technological competition affects the distribution of real and relative economic gains, it is at the same time of highest relevance for security policies, and it is closely linked to geopolitical and ideological aspects of rivalry. All this is evident in the intense debate over the use of Chinese components in the development of 5G networks and other future-oriented and critical infrastructure.

U.S. policymakers mainly emphasize the risks of espionage or sabotage in this context. This may hide a more important aspect that European debates increasingly mention: technology is not value neutral. Technological competition is strongly linked to the political-ideological sphere—the competition between liberal-democratic and authoritarian concepts of society. This is all the more the case when technological developments touch on fundamental questions of political order—as is particularly true for data acquisition and use, artificial intelligence, or biotechnology.

In the United States, technological rivalry is also seen in the light of the status contest—the competition about which nation will be number one in the world. In European discussions, the “status” question plays a lesser role: Europe is no longer in the race for overall number-one status, and Europe’s perception of what is at stake is, therefore, based on somewhat different considerations of risks, threats, and opportunities. As far as technology is concerned, Europe tends to focus more on its own competitiveness as well as its technological and hence political and strategic autonomy. In the words of French president Emmanuel Macron, Europe must not depend on American or Chinese technologies, and it must be able to guarantee that the data of its citizens will not be “stored in a space that does not come under its jurisdiction” (Macron 2020). Moreover, European decision-makers and pundits are increasingly looking at what could be called the “governance content” of new technologies. This relates to the concept of “digital spheres of influence” (Schulze and Voelsen 2020), and questions the ways in which the development and export of technologies that allow new forms of social control could be both used by authoritarian regimes within their own borders and simultaneously deployed to promote the spread of illiberal models of government in other parts of the world.

Concerns about ideological influence are certainly not limited to one side. It would be worth discussing and finding out whether decision makers in Western democracies actually understand—or, probably, underestimate—the Chinese leadership’s threat perception with regard to liberal values and world views. From the outside, at least, it seems that individual human rights, the rule of law, and liberal democracy continue to have strong appeal in relevant parts of China’s society. If this is true, it may explain the nervousness of China’s Chinese Communist Party and state leadership with regard to Hong Kong, their apprehensions with regard to potential “color revolutions”, and their efforts to find technocratic solutions to securing Party rule and, ideally, a “harmonious society.” Europeans (including this author) tend to find these apprehensions exaggerated. But things may look different from Beijing.

The Sino-American rivalry clearly extends beyond the bilateral relationship. Globally, it impacts the work of international organizations, economic and societal connectivity, and regional geopolitics. While the Trump government withdrew from or undermined existing multilateral institutions, China has been building new international fora and organizations that correspond to Beijing’s own ideas of world order. China also increasingly contributes to and participates in the activities of the United Nations (UN) and its sub-organizations. From this author’s perspective, China’s stronger buy-in into the UN system is a good thing. But it is not a remedy to the weakening of multilateral organizations. The combination of U.S. withdrawals from international organizations and regimes, and Chinese as well as Russian assertiveness within them, has actually led to more than one blockade.

## The COVID-19 effect

The COVID-19 crisis has not mitigated the rivalry between the U.S. and China. On the contrary, it has, initially at least, sharpened the ideological aspects of the competition. This is particularly so since China began to present its Communist-party led political system as superior to democratic models in dealing with such a crisis. From a European perspective, the picture looks a bit different: there is wide acknowledgement in European as well as in wider international political, academic, and media discourse that China got many things right in fighting the disease, and that it got the virus under control much earlier than other countries. But more inconvenient questions are also being raised, particularly regarding whether initial attempts to conceal the outbreak of the virus in China were also as much linked to the nature of the political system as the government’s later ability to successfully enforce effective disease-control measures.

For the time being, it is not yet clear whether China’s attempts to increase its soft power through well-staged aid deliveries (“mask diplomacy”) to countries around the world, including EU member states like Italy, have actually succeeded—or whether China’s simultaneously more assertive posture in national (Hong Kong, Xinjiang) and international settings (border clashes with India, conflicts in the South China Sea, “wolf diplomacy” in Europe and other parts of the world) have instead undermined its reputation. The results may well be different in different parts of the world. And China has obviously been learning that it is better for its own standing—particularly in the Global South—if it participates in global initiatives such as COVAX (aimed at allowing global equitable access to COVID-19 vaccines) instead of only relying on its own bilateral outreach to partner countries.

In contrast, the United States under President Trump did not even attempt to improve America’s global image in the crisis by, for example, coordinating an international response, or by supporting the respective efforts of other players like the EU, the UN, or the World Health Organization (WHO). Significantly, the U.S. did not become part of the COVAX initiative in 2020, whereas China eventually joined. International trust—a main element of soft-power—in the United States had already dropped since the start of the Trump presidency, but it decreased further after the outbreak of the pandemic. One of the lessons of 2020, however, perhaps for China’s leadership too, was that the distribution of soft power among different states is not a zero-sum game. Recent polls in selected countries suggest that the sharpened rivalry between the two states and their respective responses to the pandemic have neither helped the U.S. nor the Chinese leadership to gain in international reputation (Silver et al. 2020). Rather, both seem to have lost. We can expect a reversal of attitudes towards the U.S. after the Biden administration takes office, particularly if it engages decidedly and constructively on global issues like health and climate change. As of the time of finalizing this paper, however, before the inauguration of Joe Biden as the 46th U.S. president—this remains a hypothesis.

## Consequences for Europe

The Sino-American rivalry directly and indirectly affects the European Union and its member states. At the same time, Europe’s view of China has also become more critical. With different nuances, this applies both to the official political discourse—as reflected in the above-cited EU strategy paper on China (2019) or other documents—and to public opinion.

### Critical views and a strong interest in healthy relations

Human rights issues figure particularly strongly in the public debate on China: European societies generally believe in the universality of human rights and do not see them as internal affairs. International supply chains have increasingly become a focus in this regard. Concerns are growing about the conditions under which raw materials or manufactured goods may have been produced in China, particularly in cases involving European companies. This has repeatedly become a subject of parliamentary debates and hearings in national parliaments as well as the European Parliament, all of which will have a say on the eventual ratification of a China-EU investment agreement. Concerns about rule of law figure particularly heavily among people from business or academia who are generally seen as “pro-China” and have a strong interest in maintaining and intensifying relations on all levels. The apparent link between the arrest and subsequent detention of two Canadian citizens in 2018 following the arrest of a top Chinese businesswoman in Canada has aroused fears that Europeans travelling or working in China may be personally at risk if a conflict were to erupt between Beijing and their own governments. More recently, Chinese attempts—or what is regarded as such—to censor the international debate on China by declaring certain subjects off limits or imposing specific readings of history on universities or museum exhibits abroad have been striking a negative note in European countries (Urbach 2020). The introduction of a new security law in Hong Kong was generally seen both as a clamp-down on political freedoms, and as a unilateral change of the status agreed under the Sino-British Joint Declaration of 1984. It has therefore raised doubts about China’s commitment to international agreements—and hence to international rule of law. One can find more nuanced views on this subject in think tank publications or quality media (Rudolph 2020; BBC 2020), but the general reaction to the Hong Kong security law was negative all over Europe. The issue may have gained particularly high attention because it came in the context of what was seen as a generally more authoritarian trajectory inside China, the prospect of China exporting its model of authoritarian rule into third countries, and increasing Chinese assertiveness and more aggressive diplomacy—be that in the South China Sea, on the China-India border, or in other disputes. Verbal attacks against public figures in Sweden and trade sanctions against Australia have been regarded as test cases for how China might bully democratic countries in the event of future political controversies (Hökmark and Oksanen 2020).

European policy makers and media are aware of the fact that China sees many of these issues differently. Europeans tend to regard China as a competitor and, to a lesser extent, as a partner (Bertelsmann 2020)—but certainly not as an enemy. What various polls clearly show, however, are increasing worries over the political development path that China is taking.

At the same time, Europe is interested in stable and healthy relations with China. This is because of its direct political and economic interests with China as well as its vision for the future of multilateral cooperation and world order. In contrast to discourse in parts of the U.S. political spectrum, “decoupling”—severing technological, scientific, or economic ties with China—is clearly not an option for the EU. What has been occurring instead are more coordinated attempts among the EU and its member states to step up to the challenge of China’s growing influence in the world and Beijing’s attempts to spread its own political model, while taking care not to undermine economic and technical cooperation, interdependence, or the foundations of multilateral order. Under the paradigm of the strategic rivalry with China, American and American-influenced policy debates tend to emphasize—or, in the view of this author, over-emphasize—the vulnerabilities that go along with interdependencies. The stabilizing effects of such relations, especially the interest even of antagonistic powers in maintaining mutually beneficial relations, are too often forgotten. Europe, similar to countries in the Asia–Pacific region such as India, Japan, ASEAN states, or Australia, cannot even imagine abandoning economic exchanges and interdependencies with China. But all of these states are increasingly aware of the need to avoid unilateral dependencies.

Europe has begun to develop its own instruments for a more coordinated China policy. This includes a broadened perspective on economic interactions—a perspective that does not abstract away from their political and geopolitical aspects, particularly regarding Chinese foreign direct investment (FDI) in critical infrastructure or critical technologies. The EU and its member states have long shown strong interest in finalizing an investment agreement with China. But there is less tolerance today than a few years ago for allowing Chinese capital to invest in technologies or services in Europe that would not be open for European investment in China. Consequently, member countries have enacted EU-inspired investment screening processes. Even European business leaders—the strongest voice defending good relations with China—are usually in favor of such measures, seeing them as a way to bring more balance into China–Europe economic interaction. European businesspeople see with some concern that China itself may opt for some form of selective decoupling. As the secretary general of the German Federation of Industries pointed out in an op-ed, there is also increasing concern that China may attempt to use supply chain dependencies as a means to exert political pressure on other countries (Lang 2020), whether in Europe or elsewhere in the world.

The view of German business is particularly significant here, not only because Germany is the biggest economy in the EU. Germany under Chancellor Angela Merkel (2005-present) has been a strong defender of economic cooperation and political dialogue with China, and it has therefore occasionally been accused of being “soft” or “wavering” on China (Barkin 2020). In fact, Germany, as well as France and others have realized that they need to make a stronger effort to coordinate policies towards China within the EU and avoid the impression that Europe is acting under a common trade regime but politically fragmented in dealing with China. This is very much in line with an increasing realization, in Germany as well as in other EU countries, that “Europe,” or more precisely EU-unity, has become the prime *national* interest in the current era of renewed great power competition and of global challenges that no single country can cope with on its own (Bagger 2020).

Given its political and business model, Europe remains loath to engage in great power conflict itself, but it has realized that it needs to strengthen its own capabilities and increase what is now more or less interchangeably called European “strategic autonomy” or sovereignty. In essence, strategic autonomy is about defining one’s own priorities and making one’s own decisions in all foreign and security policy matters, as well as possessing the political, material, and institutional resources to carry them through. Autonomy—or sovereignty—does not mean autarchy, and it does not mean a rejection of alliances. Rather, an autonomous actor decides on her own about the alliances and partnerships she wants to establish, and she does so on the basis of her values and interests. There is little doubt among the majority of Europeans that the United States will remain the alliance partner of choice—despite the more fundamental differences, even on values and visions of international order, that have marked the relationship in the era of President Trump (Lippert et al. 2019; Borrell 2020). What Europeans have learned in these past few years is that Europe—in the words of German Chancellor Merkel—will have to rely more on itself.

The EU has indeed been able to strengthen its cooperation on security and defence, and to devote more common resources for this purpose. However, jointly defining geopolitical interests and priorities remains a challenge. Foreign observers as well as European critics—who generally want deeper European integration, including on foreign and security policies—occasionally overlook the complex realities of the EU. While it is a supra-national political actor in many realms (particularly with regard to trade and regulation), it is also a Union of 27 sovereign states which all have their own, not necessarily congruent interests. And it remains very much a learning institution the weaknesses and strengths of which become particularly apparent in times of crises. Arguably, the EU has fared much better during the COVID-19 crisis than during the financial crisis of 2008/2009. Despite some initial “my-own-country-first” reactions, the EU proved resilient during the pandemic in 2020 and managed to visibly strengthen its common action. Most importantly, it has been able to patch together a 750-billion Euro rescue fund, based on common borrowing (which Germany and other more fiscally conservative countries had previously rejected) and connected to an internal rule-of-law mechanism. It has also managed to avoid a harsh Brexit by negotiating and agreeing upon a free-trade agreement with the United Kingdom. At the same time, European actors have been increasing their practical commitment to strengthening multilateral cooperation and organizations. From a European perspective, global responses to the COVID-19 pandemic—such as the “ACT (access to COVID-19 tools) accelerator” or the COVAX initiative—demonstrate that multilateralism works, even in a crisis and even in the shadow of sharp great-power rivalries.

## Outlook

EU leaders and the European public do not expect the rivalry between the United States and China to disappear any time soon. The Biden presidency will likely not be “soft” on China. However, it will be more diplomatic, see to it that differences do not turn into open conflict, and seek to manage what Joseph Nye (2020) calls the two nations’ “cooperative rivalry” in a careful way that does not threaten common global interests. At the same time, the Biden administration will also expect from Europe that it support U.S. policy towards China, not least on issues like 5G technology, trade rules, or alliance systems in the Indo-Pacific. This will be easier for Europeans than it was in the era of Donald Trump, who seemed to see the European Union not as a partner but as an adversary, and at one point even called it “worse than China.” We may see a historical dialectic of sorts at work here: Europe did in many ways share the Trump administration’s criticism of Chinese policies and practices, but it was also critical of that administration’s undiplomatic handling of China, and it demonstrated its own interest in maintaining strong cooperative relations with China—not least through high-level summits with the Chinese leadership. Europe will likely seek much closer coordination on China with the Biden administration. This may in turn raise suspicions in China and add some conflictual elements into the China–Europe relationship. The well understood self-interests of both the EU and China demand that such disputes are dealt with in an institutional setting, on the basis of mutual respect and international law, and without losing sight of common global challenges.

In Europe, both positive and negative experiences throughout the pandemic have underscored the view that China needs to remain a global cooperation partner despite the “systemic” aspects of rivalry. In 2019, when the EU strategy paper on China was published, climate change was the most obvious subject for global cooperation. It still is, and China’s recent announcement that it will reach carbon-neutrality by 2060 is important and welcome. The COVID-19 pandemic and its economic fallout have, atop of this, underscored the importance of cooperation on global health issues, debt relief, and economic recovery (Mair et al. 2020). With all of this, there are significant conceptual and political differences, particularly between EU-Europe and China, about what multilateralism actually is. Also, multilateral cooperation is no panacea to avoid conflict; nor does it as such mitigate political and geopolitical rivalry. But it certainly can help to manage and civilize rivalries.

## Footnotes

1 The first part of this paper draws partly on Lippert and Perthes (2020).
